# A Training Game for Students Considering Family Medicine: an Educational Project Report

**DOI:** 10.25122/jml-2019-0056

**Published:** 2019

**Authors:** Inès Van Rossem, Dirk Devroey, Kristien De Paepe, Francis Puttemans, Pascale Petit, Sandrina Schol, Sander Deridder, Jan Vandevoorde

**Affiliations:** 1. Department of Family Medicine and Chronic Care, Vrije Universiteit Brussel, Brussels, Belgium; 2. Department of Pharmaceutical Sciences, Vrije Universiteit Brussel, Brussels, Belgium; 3. Study Guidance Centre, Vrije Universiteit Brussel, Brussels, Belgium,; 4. Department of Chemical Engineering, Vrije Universiteit Brussel, Brussels, Belgium

**Keywords:** Medical education, Skills training, Communication, Simulation based medical education, Clinical competence

## Abstract

The Groningen Institute Model for Management in Care Services aims to prepare medical students for their complex tasks as family physicians, based on the CanMEDS framework. Although initially developed for pharmacy students, the present paper reports on the eight-year experience with GIMMICS for family physician students at the Vrije Universiteit Brussel.

The Groningen Institute Model for Management in Care Services is a training game that simulates real-life situations in a structured and supervised setting. It offers students the possibility to practice clinical, practical, and communicational skills. Students install and manage their group practices, hold consultations with simulated patients, participate in several assignments and collaborate with pharmacy students. Feedback sessions showed that the training game is well-received by the students. A self-assessment questionnaire comprised of 23 questions on significant aspects of the seven CanMEDS roles showed significantly higher scores at the end of the game for 17 questions (p<0.05, Wilcoxon signed-rank test ).

GIMMICS is a valuable linking pin between the different learning methods in medical education and clinical practice, helping students to improve themselves in the CanMEDS roles. However, simulation-based medical education requires significant time and resource investment.

## Introduction

The Groningen Institute Model for Management in Care Services (GIMMICS) has long-standing expertise in the training of pharmacy students in the Netherlands [1]. It is a training game in which students can apply their knowledge, attitude, and skills in an integrated way and a safe learning context. Last year, pharmacy students collaborated in small teams to run their fictional pharmacy on the premises of the university campus [2]. This paper describes the implementation of this model in the training of medical students aiming to specialize in family medicine at the Vrije Universiteit Brussel.

The CanMEDS Physician Competency Framework describes the knowledge, skills, and abilities that physicians need for better patient outcomes. It is based on seven roles that all physicians need to acquire in order to become better doctors: Medical Expert, Communicator, Collaborator, Leader, Health Advocate, Scholar, and Professional [3]. Although many models have been set up to teach and learn these skills, many students do not feel they fully master them at the end of their curriculum [4].

Training of family physician students consists of a well-balanced mix of training in medical skills, attitude, and knowledge. Apart from theoretical courses, medical and communication skills are improved by training in real-life conditions in hospitals and primary healthcare practices. It is unavoidable that students do not have the same opportunities to learn and practice the same pallet of skills in each of the training practices. This variability in trainee’s posts does not only exist in family practices but also in hospitals [5]. An essential aspect of learning environments in clinical settings is the type and quality of opportunities they offer to practice important, highly demanding, or responsible tasks. Rotem et al. argued that emphasis should be given to the creation of supportive and well-organized learning environments in clinical settings [6].

Due to the continuously increasing amount of medical information, it is becoming more and more challenging to stay up to date. This makes it hard for medical schools and teachers to adapt their curricula accordingly, and it compels them to make pedagogical shifts: more use of small-group sessions, increasing self-directed learning, more independent research, the use of laboratory skills, and others [7]. 

In addition, some students feel that they are not fully prepared for real-life practice at the end of their studies [7-9]. Simulation-based medical education (SBME) could be a solution to these problems [7,10]. The effectiveness of simulation in teaching clinical knowledge, procedural skills, teamwork, and communication, as well as the assessment, are reported by Okuda et al. [7].

As a result of the good experiences of pharmacy students and their teachers with GIMMICS, the Department of Family Medicine and Chronic Care of the Vrije Universiteit Brussel decided to introduce the GIMMICS model in the training of medical students aiming to specialize in family medicine. This paper describes the characteristics and the nine-year experience with the training game.

### Description and assessment of the game

The game aims to prepare medical students for their complex tasks as family physicians, based on the CanMEDS framework, by addressing and integrating each and all of the seven roles (Table 1).

**Table 1:** Realization of the CanMEDS roles in GIMMICS.

CanMEDS roles		Realization in GIMMICS
**1. Medical Expert**	• Clinical reasoning and decision-making • Perform a patient-centered clinical assessment and establish a management plan • Plan and perform procedures and therapies for the purpose of assessment and/or management	• Consultations and home visits • Short-term assignments
**2. Communicator**	• Establish professional therapeutic relationships with patients and co-workers • Share health care information and plans with patients • Document and share written and electronic information about the medical encounter	• Consultations and home visits • Collaboration with pharmacists
**3. Collaborator**	• Work effectively with physicians and other colleagues	• Collaboration with pharmacists • Installation and management of the practices
**4. Leader**	• Manage career planning, finances, and human health resources in a practice • Time management • Contribute to the improvement of health care delivery in teams, organizations, and systems	• Installation and management of the practices • Long-term assignments
**5. Health Advocate**	• Respond to the needs of the communities or populations they serve by advocating with them for system-level change in a socially accountable manner	• Long-term assignments
**6. Scholar**	• Engage in the continuous enhancement of their professional activities through ongoing learning • Integrate best available evidence into practice	• Long-term assignments • Collaboration with pharmacists
**7. Professional**	• Demonstrate a commitment to patients by applying best practices and adhering to high ethical standards • Demonstrate a commitment to the profession by adhering to standards and participating in physician-led regulation	• Consultations and home visits

GIMMICS aims to simulate real-life situations in a structured and supervised setting, thereby avoiding the heterogeneity (variability in the population, pathology, organization, and others) existing between different trainee posts. However, it should be emphasized that GIMMICS is organized in addition to internships in family practices and does not replace them.

GIMMICS is organized in the last year of the basic medical training for family physician students. They work in small teams to run their practice for two weeks in a fictional town on the premises of the university. Before the training, the participating students receive all the information needed to understand the aim of the training, the practical organization, and arrangements. GIMMICS is supervised by one coordinator supported by a team of staff members of the Department of Family Medicine and Chronic Care. The students are grouped into proportionally divided training practices considering gender balance and prior evaluations on knowledge, attitude, and skills, thus attaining well-balanced heterogeneous groups. For each cohort, three training practices are established, and each training practice consists of six to seven students.

### Installation and management of the practices

Both installation and management of the practices help students to grow in the CanMEDS roles of leader and collaborator.

Each practice receives a classroom area, two mobile phones, a computer with an electronic medical record (EMR) installed, and a printer. In a prior 3-hours training, the students are instructed how to use the EMR. Each practice has a fictional budget to ‘buy’ medical equipment for their practice. At the end of the first day, they set up the practices and the waiting rooms with the material they ‘bought’. Students have to manage their group practice. They receive a schedule for consultation and home visits, which, when necessary, they must adapt to unforeseen circumstances (e.g., emergencies, absence of one of the doctors). They need to set up a rotation system for out-of-hours services and emergency calls during the nights and weekends. For practical reasons, the out-of-hours services consist of telephone calls that can be handled as such. Students are also responsible for the stock management of the practice.

### Consultations and home visits with simulated patients

The clinical work with simulated patients (SPs) helps students master the role of a medical expert, communicator, and professional.

There is evidence that SPs are valuable tools for training communication skills and technical skills [11], and they are well received by students [12]. During the training game, real-life clinical situations are simulated with the use of SPs in consultations and at-home visits. These SPs are trained to portray clinical situations consistently on repeated occasions. Briefing and training of the SPs are of the utmost importance. They receive oral and written directives before the start of the game and are briefed once more by the coordinator right before the start of their session. During consultations, students are coupled two-by-two: one student observes and gives feedback to the other student, and vice versa. This kind of peer-review is a learning moment for the physician-student as well as the observatory-student. Because of their role as a supervisor, it is of the utmost importance that the students are informed about the learning objectives before the start of the training game [13]. Students see on an average day about five patients. Each consultation session is followed by a feedback moment supervised by a staff member that is fully trained in supervision and giving feedback. Clinical supervision of consultations stimulates the delivery of good quality care and improves safeguarding standards of care, the development of professional expertise, and the delivery of quality care [14]. In our GIMMICS setting, staff, as well as students, act as supervisors and are both able to give direct guidance on clinical work, link theory and practice, engage in joint problem-solving, and give feedback.

Students are instructed to make at least two videotapes of consultations of their choice. They analyze one video fragment using the Calgary-Cambridge Referenced Observation Guides as a practical teaching tool to delineate and structure the skills which aid communication between patients and physicians [15]. In addition, the videos are used by the staff members to give feedback in the group on communicational skills using the common ground instrument [16]. The use of videotapes is beneficial, but unfortunately, it is also very time-consuming [17].

### Long-term and short-term assignments

Students participate in two long term assignments during the entire timeframe of the training game. In this way, they train their skills as a leader, health advocate, and scholar.

First, each practice engages in a quality improvement project (QIP), as a common theme throughout their GIMMICS period, using a Find, Organise, Clarify, Understand, and Select - Plan-Do-Check-Act (FOCUS-PDCA) approach [18,19].

Secondly, students must complete the GIMMICS chapter of their portfolio [20,21]. The students reflect on their learning agenda, on an analysis of a video fragment, and describe a doctor’s educational need experienced during the game, including the associated literature study [22]. With the use of this portfolio, it is aimed to stimulate the concept of lifelong learning.

Students also receive several short term assignments on specific topics enhancing their skills as a medical expert. First, they are asked to fill in written exercises about procedures such as blood sampling and laboratory investigation, dermatology, and ECG.

The second type of short term assignments consists of filling out administrative paperwork (such as death certificates, applications for handicap allowance, and disability forms). Furthermore, a structured essay is made on the development of a care plan and the associated social map for a palliative patient. Finally, specific clinical skills such as taping, reanimation, performing a 12-lead ECG, and spirometry are covered.

### Collaboration with pharmacists

During GIMMICS, the training for family medicine students runs simultaneously with the training for pharmacy students offering the opportunity to encourage interprofessional collaboration. This stimulates the students in their role as a scholar, collaborator, and communicator.

For this purpose, medico-pharmaceutical meetings are organized. Medical students also receive courses and exercises on medical prescriptions and prescriptions for compounding, and they have the opportunity to observe the preparation of a compounded drug. Finally, groups of family physicians and pharmacists reflect together on rationalizing medication schemes for patients with polymedication.

### Assessment of the students

For the overall assessment of knowledge and skills during the entire medical program, 4-stages Miller’s pyramid is used [23]. GIMMICS is a good tool to test for level three. As the existing assessment model for pharmacy students was not applicable for medical students, an adapted multisource model was developed (Table 2).

**Table 2:** Assessment of the students during GIMMICS

Formative assessment through feedback	Summative assessment
By supervising clinicians on therapeutic and diagnostic handling, on clinical skills and communicational skills: • Through direct observation of consultations • Through video-review	Written short-term assignments: • multiple-choice questions and short-answer questions on specific topics • evaluation of administrative tasks • structured essays on specific topics in chronic care
By simulated patients filling in a survey on the students’ ability to gain trust, patient satisfaction and interpersonal behavior	Quality improvement project: presentation on how they conceived and implemented their project
By peers on all aspect of the consultation through direct narrative feedback	Portfolio
	A rating on professional behavior by all the staff members: • on how they handle their professional work • on how they interact with patients, colleagues and staff members • on their dedication to the game

The first method of formative assessment is performed by supervising clinicians. Observation by a supervisor and feedback from the teacher to the learner are well-known beneficial educational activities [24-27]. Secondly, consultations are also scored by SPs. To our knowledge, there are no clear standards concerning effective feedback training for SPs [28]. Finally, students also give direct narrative feedback to their peers.

The classification of students in a summative way is based on the written short term assignments, presentations of the QIP’s, scoring of a portfolio [29], and scoring of their professional behavior.

### Perception and evaluation of the game

Previous GIMMICS runs were evaluated in three ways. Firstly, a plenary feedback session with the students, which was held during the nine editions the program has been running. Secondly, a self-assessment of the students, before and after the game, was introduced in the two most recent runs of the program. Finally, each edition was reviewed by all the participating staff members.

On the final day of GIMMICS, a plenary assessment session of two hours with students and staff members is held to inquire about the satisfaction of the students. This gives students the possibility to offer feedback on every aspect of the training game.

Past sessions revealed that the training is generally well-received. Students appreciated that they could take full responsibility in an almost real-life consultation and practice setting without the danger of harming a patient. The newly gained independence and responsibility, inherent to the game, is considered to be both instructive and challenging for the students. The fact that the staff, as well as students, act as supervisors during the consultations and are both able to give direct personal guidance and feedback on the entire consultation process, is highly appreciated by the students.

In general, the students asked for a longer duration of the game and preferred that a more significant part of the game time would be spent on consultations, including more video consultations and personal feedback. This is in line with the results of the study by Longman et al. [27]. Students also appreciated their teamwork and collaboration with the pharmacy students.

During the last two editions of GIMMICS, a self-developed questionnaire was filled out by the students before and after the game, scoring themselves on a 5-point Likert scale. This anonymous survey comprised 23 questions on significant aspects of the seven CanMEDS roles (Annex). The purpose of this questionnaire is to determine students’ progress in a more quantitative way. Comparison before and after showed higher scores at the end of the game for every single question. A Wilcoxon signed-rank test showed that this progress was significant (p<0.05) for all but two questions about the role of professional (Table 3).

**Table 3:** Assessment of GIMMICS by the students through a self-assessment survey in the last two runs of the game.

	Question	Neg (N)	Mean Rank	Pos (N)	Mean Rank	P-value*
**1.**	Do you take the socio-demographic factors of a patient into account when making a diagnosis?	1	17.00	16	8.50	.002
**2.**	How would you score yourself on drawing out a diagnostic landscape after the anamnesis?	1	19.00	19	10.05	<.001
**3.**	How would you score yourself on the formulation of a medical policy?	1	10.00	23	12.61	<.001
**4.**	How would you score yourself on your ability to consult in a logical and structured manner?	6	9.75	20	14.63	.002
**5.**	How well do you know the indications of supplementary examinations?	0	0.00	19	10.00	<.001
**6.**	How would you score yourself on your ability to build a good patient-doctor relationship?	2	8.00	14	8.57	.004
**7.**	How would you score yourself on your listening skills?	3	10.83	14	8.61	.033
**8.**	How well do you explore the ICE (Idea’s , Concerns, Expectations) of the patient during consultation?	0	0.00	26	13.50	<.001
**9.**	Do you think that you can communicate a diagnosis and treatment to a patient in a simple and clear manner?	1	19.50	20	10.58	<.001
**10.**	How well do you transfer patient related information to other involved health care professionals?	3	8.50	17	10.85	.001
**11.**	To which degree do you consider yourself as being a team player?	0	0.00	16	8.50	<.001
**12.**	How well do you know the social landscape and where to find it?	1	19.50	30	15.88	<.001
**13.**	How would you score yourself for time management?	2	13.75	24	13.48	<.001
**14.**	How well is your knowledge of ICT (necessary to perform your work as a physician?	4	10.50	16	10.50	.010
**15.**	How well do you abide by agreements made with co-workers?	3	10.00	20	12.30	<.001
**16.**	How well can you develop a preventive health care program for a family practice?	1	10.00	24	13.13	<.001
**17.**	How would you score yourself on your ability to quickly find relevant information in medical literature?	2	7.50	16	9.75	<.001
**18.**	How would you score yourself on formulating a correct PICO (Patient Intervention Comparison Outcome)?	5	11.00	19	12.89	.003
**19.**	How would you score yourself on critical revising and interpretation of scientific research?	2	5.00	16	10.06	<.001
**20.**	How would you score yourself on working evidence based?	0	0.00	19	10.00	<.001
**21.**	How well do you stimulate patients in taking joint responsibility for their own health?	4	11.50	17	10.88	.007
**22.**	To which degree do you consciously handle your emotions during your role as a physician?	5	13.90	17	10.79	NS
**23.**	How would you score yourself on your ability to separate work from private?	4	9.50	12	8.17	NS

After each edition of the game, a short plenary feedback session is held with all the participating staff members, reviewing the general course of the game. Past sessions indicate that all staff members are fully convinced that the game is a profitable innovation made to the curriculum. Their main concern, on the other hand, is keeping the balance between creating a rich game setting and not overcomplicating the management of the game. Practical problems that arose during the latter edition are being addressed and will be taken into account for future runs.

## Discussion

GIMMICS is a training game in which students run their own fictional family practices. It is a rather new implementation of SBME. Closely resembling a real-life internship, it offers some additional advantages. The main benefit being that students carry full responsibility for the entire consultation from beginning to end without any guidance from a supervisor during the consultation. It allows students to grow in and integrate the seven CanMEDS roles. An evaluation of the game by a self-assessment survey of the students on the CanMEDS roles revealed that they score themselves significantly better after the game than before, suggesting that GIMMICS is a useful educational tool. In addition, oral feedback sessions revealed that the game is well received by the students. Notably, the numerous feedback sources were highly appreciated.

It should be mentioned, however, that GIMMICS requires time and resource investments. In particular, the direct observation and video review of consultations and the accompanying individual feedback are time-consuming. Moreover, the peer to peer feedback was not always supervised due to staff shortage, making its quality a concern for the future. SBME is a complex service intervention that needs to be planned and practiced with attention to organizational contexts [30]. Fitting rooms for practices, waiting rooms, rooms for feedback sessions and others, require logistic support from the university. The remuneration of SPs and the purchase of the equipment for the simulated practices represent a financial cost. A final practical limit is the number of students that can participate per cohort. It seems feasible to work with larger numbers of students, but it would demand careful planning beforehand.

The GIMMICS model offers future opportunities to build a broader healthcare community within the game by including other health care workers. For example, students in physiotherapy or dietetics could join in and open their own fictional practices in the proximity, thus further stimulating the interdisciplinary collaboration.

Continuation of the self-assessment in the future and implementation of qualitative analyses of the feedback sessions might provide a more solid basis to adapt the game accordingly.

## Conclusion

The GIMMICS model allows medical students to train different aspects of the CanMEDS roles in a well-protected simulated environment and offers an opportunity for active and reflective learning. It is a valuable linking pin between the different learning methods in medical education and clinical practice. It smoothes the path from practice training to community healthcare work and integrates academic knowledge and social competencies. It should be said that GIMMICS requires time and resource investments, which should be taken into account before attempting to organize the game. Therefore, the key messages are:

• GIMMICS is a simulation-based training game where medical students set up and work in medical practices.
• The aim of GIMMICS is to prepare students for their task as future family physicians by addressing and integrating the seven CanMEDS roles.
• GIMMICS is a valuable linking pin between theory and clinical practice.

## Conflict of Interest

The authors confirm that there are no conflicts of interest.

## EVALUATION OF GIMMICS; PRE / POST SELF-ASSESSMENT

This is an evaluation of GIMMICS itself as a learning tool. Everything is handled completely anonymous.

Individually chosen random code:

### MEDICAL EXPERT

1. Do you take the socio-demographic factors of a patient into account when making a diagnosis?

1 2 3 4 5

2. How would you score yourself on drawing out a diagnostic landscape after the anamnesis?

1 2 3 4 5

3. How would you score yourself on the formulation of a medical policy?

1 2 3 4 5

4. How would you score yourself on your ability to consult in a logical and structured manner?

1 2 3 4 5

5. How well do you know the indications of supplementary examinations?

1 2 3 4 5

### COMMUNICATOR

6. How would you score yourself on your ability to build a good patient-doctor relationship?

1 2 3 4 5

7. How would you score yourself on your listening skills?

1 2 3 4 5

8. How well do you explore the ICE (Idea’s, Concerns, Expectations) of the patient during consultation?

1 2 3 4 5

9. Do you think that you can communicate a diagnosis and treatment to a patient in a simple and clear manner?

1 2 3 4 5

### COLLABORATOR

10. How well do you transfer patient related information to other involved health care professionals?

1 2 3 4 5

11. To which degree do you consider yourself as being a team player?

1 2 3 4 5

12. How well do you know the social landscape and where to find it?

1 2 3 4 5

### LEADER

13. How would you score yourself for time management?

1 2 3 4 5

14. How well is your knowledge of ICT (necessary to perform your work as a physician)?

1 2 3 4 5

15. How well do you abide by agreements made with co-workers?

1 2 3 4 5

### HEALTH ADVOCATE

16. How well can you develop a preventive health care program for a family practice?

1 2 3 4 5

### SCHOLAR

17. How would you score yourself on your ability to quickly find relevant information in medical literature?

1 2 3 4 5

18. How would you score yourself on formulating a correct PICO (Patient Intervention Comparison Outcome)?

1 2 3 4 5

19. How would you score yourself on critical revising and interpretation of scientific research?

1 2 3 4 5

20. How would you score yourself on working evidence based?

1 2 3 4 5

### PROFESSIONAL

21. How well do you stimulate patients in taking joint responsibility for their own health?

1 2 3 4 5

22. To which degree do you consciously handle your emotions during your role as a physician?

1 2 3 4 5

23. How would you score yourself on your ability to separate work from private?

1 2 3 4 5
